# Supplementary material for: Stochastic properties of musical time series
Source: Nat Commun. 2024 Oct 28;15:9280. doi: 10.1038/s41467-024-53155-y (PMC11519375; doi:10.1038/s41467-024-53155-y)
Supplement: Supplementary file 1 — Supplementary Information Stochastic Properties of Musical Time Series [file 41467_2024_53155_MOESM1_ESM.pdf]

Supplementary Information
Stochastic Properties of Musical Time Series

Corentin Nelias[1] and Theo Geisel[1, 2, *]

[1]Max Planck Institute for Dynamics and Self-Organization
37077 Göttingen, Germany.
[2]Bernstein Center for Computational Neuroscience Göttingen
Georg August University Göttingen, 37073 Göttingen, Germany
[*]theo.geisel@ds.mpg.de

MIDI notes quantization and segmentation

To preserve the information associated with the temporal duration of notes in the time-series extracted from the raw MIDI data, each note in the sequence was quantized and segmented according to a grid of unit duration of 1/12th of a quarter note. An example of this procedure is given in Supplementary Figure 1, where Supplementary Figure 1a shows an example of a MIDI-signal composed of 4 notes of different pitch and durations. In Supplementary Figure 1b the quantization and segmentation procedure was applied. The pitch of the notes was left unchanged, but the length was adapted such that the starting and ending times of the notes fit with the quantization grid. The notes were subsequently segmented according to the grid.

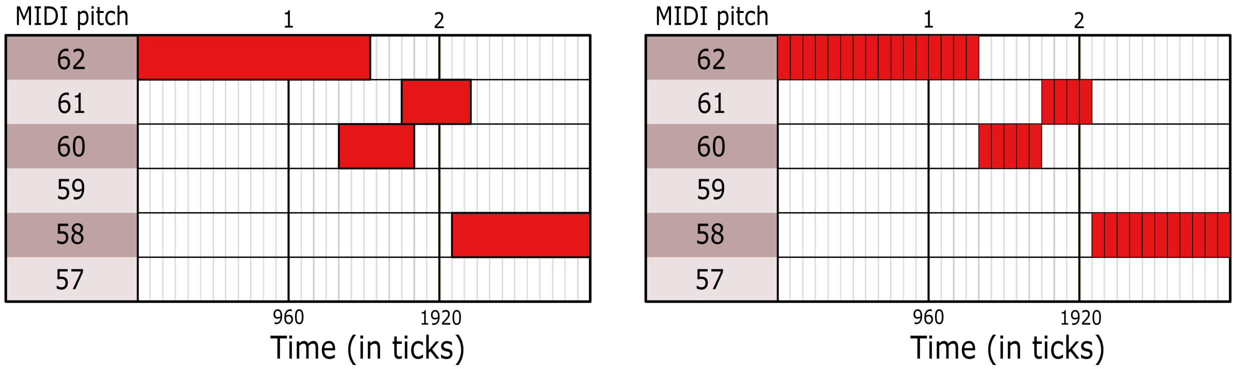

(a) Before chunking

(b) After chunking

Supplementary Figure 1. Note segmentation process. (A) presents a typical MIDI signal, (B) shows the form of this MIDI signal after processing : every note longer than the shortest time unit is replaced by a sequence of time units of the same pitch. The gray transparent lines represent the grid of time units used for quantization and segmentation.

Representation of rests

In the methods section we explained that we treated rests (absence of notes) by introducing a pitch of 0 for each time unit of silence. Even though this appears as a suitable procedure to treat rests, which has been used in previous work [1, 2], the introduction of zeros in place of missing values in a time series might create artifacts depending on the statistical distribution of the missing values.

In order to check that our results are not due to such artifacts, we applied the Lomb-Scargle periodogram to a number of pieces from our corpus and compared the results to their multitaper equivalent. The Lomb-Scargle periodogram is able to deal with missing values as it does not require uniform data sampling. It is subject, however, to a larger bias and variance than the multitaper method. The Lomb-Scargle estimation is expressed as:

$$P_{L\hat{S}}(f) = \frac{a^2}{2}(\sum_{t=0}^{N-1} x(t)cos(2\pi f[t-\tau]))^2 + \frac{b^2}{2}(\sum_{t=0}^{N-1} x(t)sin(2\pi f[t-\tau]))^2 \tag{1}$$

where

$$a = \frac{\sum_{n=0}^{N-1} x(t_n)\cos(2\pi f[t_n-\tau])}{\sum_{n=0}^{N-1} \cos^2(2\pi f[t_n-\tau])} \tag{2}$$

$$b = \frac{\sum_{n=0}^{N-1} x(t_n)\sin(2\pi f[t_n-\tau])}{\sum_{n=0}^{N-1} \sin^2(2\pi f[t_n-\tau])} \tag{3}$$

$$\tau = \frac{1}{4\pi f} tan^{-1}1\left(\frac{\sum sin(2\pi f t_n)}{\sum cos(2\pi f}\right) \tag{4}$$

The PSD estimations obtained by the Lomb-Scargle periodogram confirmed our results by revealing identical shapes (PL or PL+P), similar slopes, and cutoff times, as the multitaper estimation. See e.g. Supplementary Figure 2 and Supplementary Figure 3 for a direct comparison of the two methods for a movement of a sonata by J.S. Bach and a string quartet by Brahms.

We conclude that the replacement of rests by pitches of 0 does not change the overall slope of the PSDs.

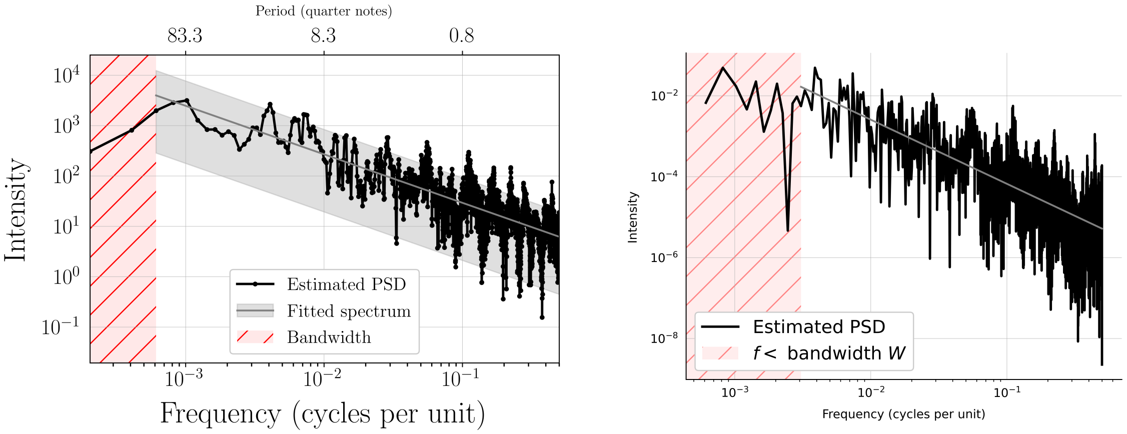

(a) multitaper PSD estimation **(b)** Lomb-Scargle PSD estimation

Supplementary Figure 2. Comparison of PSD estimation with and without missing values for the Sonata for solo Violin in G minor, BWV 1001, presto, by J.S. Bach (A) presents the PSD estimation using the multitaper method and replacing rests by a pitch of 0, (B) shows the Lomb-Scargle periodogram omitting rests. The inclusion or exclusion of missing notes as 0 pitch does not change the overall slope of the PSD. The Lomb-Scargle periodogram shows more scattering, as expected by the fact that this method does not perform any averaging.

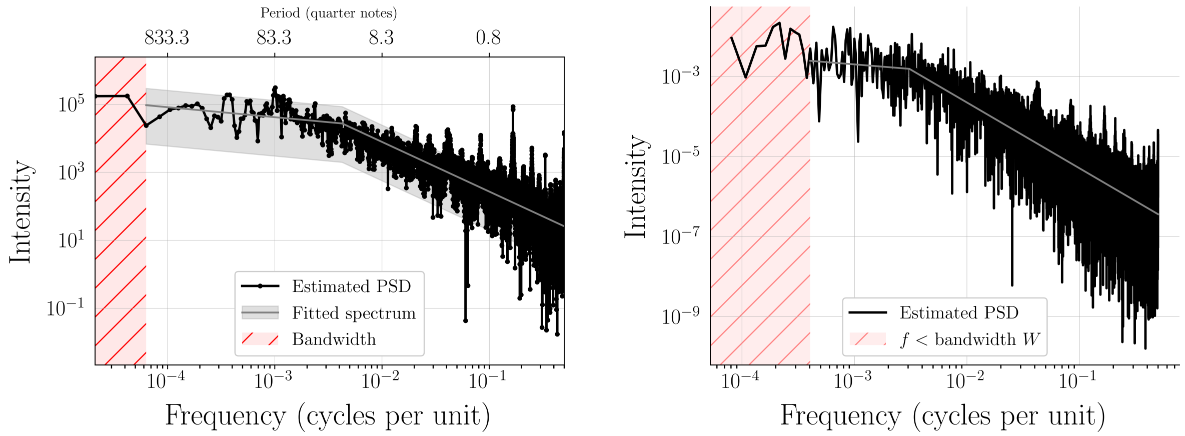

(a) multitaper PSD **(b)** Lomb-Scargle PSD estimation

Supplementary Figure 3. Comparison of PSD estimation with and without missing values for the String Quartet Op.51, No.1 in C minor by J. Brahms. (A) presents the PSD estimation using the multitaper method and replacing non-played notes by a pitch of 0, (B) shows the Lomb-Scargle periodogram omitting rests. The inclusion or exclusion of rests as 0 pitch does not change the overall slope of the PSD. The Lomb-Scargle periodogram shows more scattering, as expected by the fact that this method does not perform any averaging.

Choice of the NW product

In the main text of this article, we mention that the bias bandwidth (controlled by the NW product) can mask the existence of a plateau in the PSD. Here we provide an example illustrating this effect. Supplementary Figure 4 shows a comparison of the PSD estimation of Beethoven's 4th Symphony, Opus 60, 2nd movement for two different values of the NW product. Compared to the case with a low value of NW (NW = 3, right panel) using a higher NW value (NW = 12, left panel) yields an estimation that has a lower variance, but a broader bandwidth. Consequently, the plateau that is seen in the estimation for NW = 3 is concealed within the bandwidth for NW = 12, within which conclusions cannot be drawn as the plateau might result from the bias mixing the different frequency components. The example illustrates that an appropriate choice of NW is important to achieve accurate results. The choice of NW = 2 in this study was motivated by the fact that the bandwidth difference between the lowest possible value NW = 1 and NW = 2 is not very large, but the variance reduction going from NW = 1 to NW = 2 is considerable (see Supplementary Figure 5). NW = 2 therefore appears as a good trade-off between bias and variance.

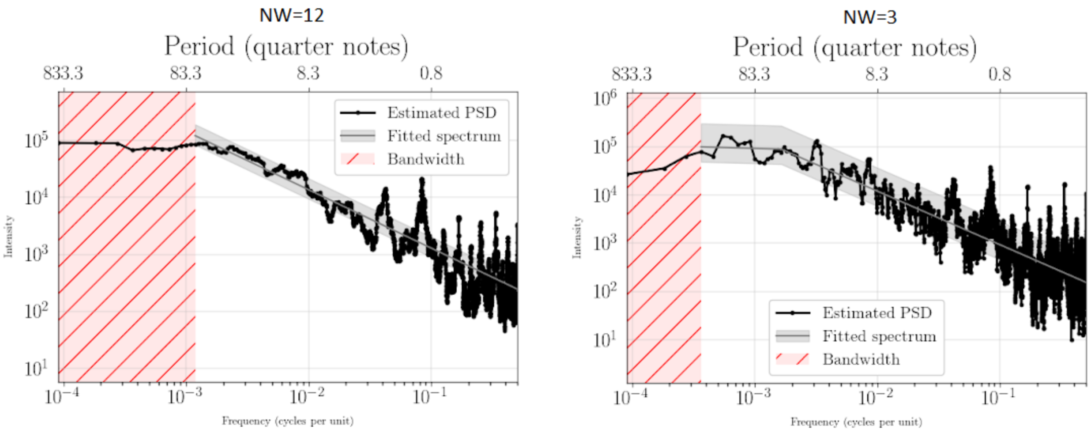

Supplementary Figure 4. Effects of the NW product on the bandwidth of the estimation and detection of plateaus. PSD estimation of Beethoven's 4th Symphony, Opus 60, 2nd movement obtained using two different NW products: 12 (left) and 3 (right). A high NW product can lead to a bandwidth so large that it conceals the plateau appearing at around period 80.

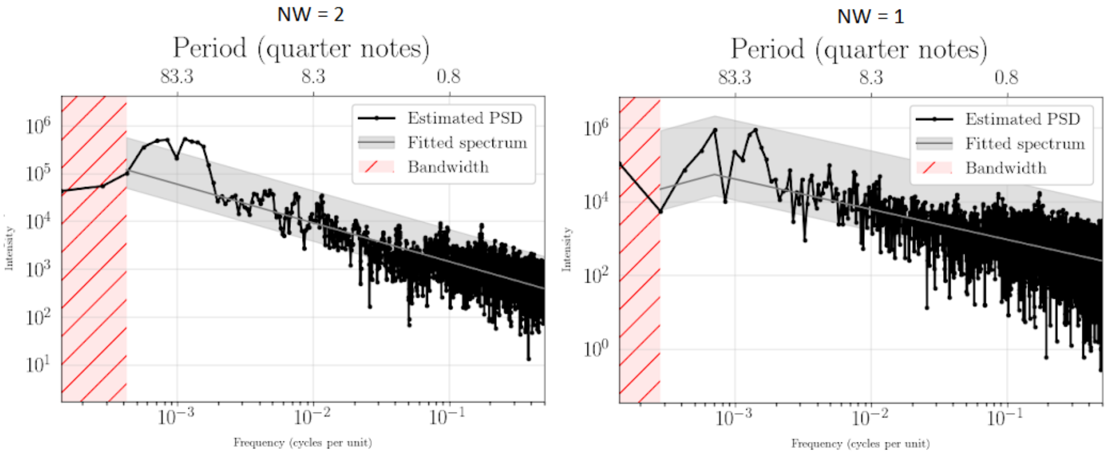

Supplementary Figure 5. Effects of the NW product on the variance. PSD estimation of David Liebman's Begin the Beguine with two different NW products: NW = 2 (left) and NW = 1 (right). Lowering the NW products reduces the bandwidth of the bias and uncovers a pateau, but considerably increases the variance.

Detrended fluctuation analysis

Power-spectral analysis, on which the current study is based, is not the only tool that can be used to study fluctuations in time series. Another popular and powerful method is detrended fluctuation analysis (DFA) [3, 4]. It even has the advantage of being readily extensible to multivariate time series, a feature which might make it appear as an ideal method for studying musical time series. It was shown, however, to be impacted considerably by the presence of periodic components in the signal[5, 6]. This may become problematic, as musical time series often show repetitions and periodic components, as emphasized by the rhythmic peaks mentioned in the main text of this article. Periodic components can lead to broad shoulders in the DFA fluctuation function, as e.g. in Supplementary Figure 6, where we compare the effect of periodic components on DFA and on the PSD of simulated pink noise. Here the PSD is able to identify the periodic component with good resolution and is otherwise unaffected, whereas the course of the DFA fluctuation function is affected over a range of window sizes much broader than the introduced periodicity.

To the best of our knowledge, the only way to correct DFA for periodic components is by manual intervention, identifying them via the PSD and then applying an appropriate filter to the original signal [6]. Since this method modifies the original signal, introduces some arbitrariness, and is not always able to reconstruct the undisturbed fluctuation function completely [6], we decided to use PSD-analysis rather than DFA.

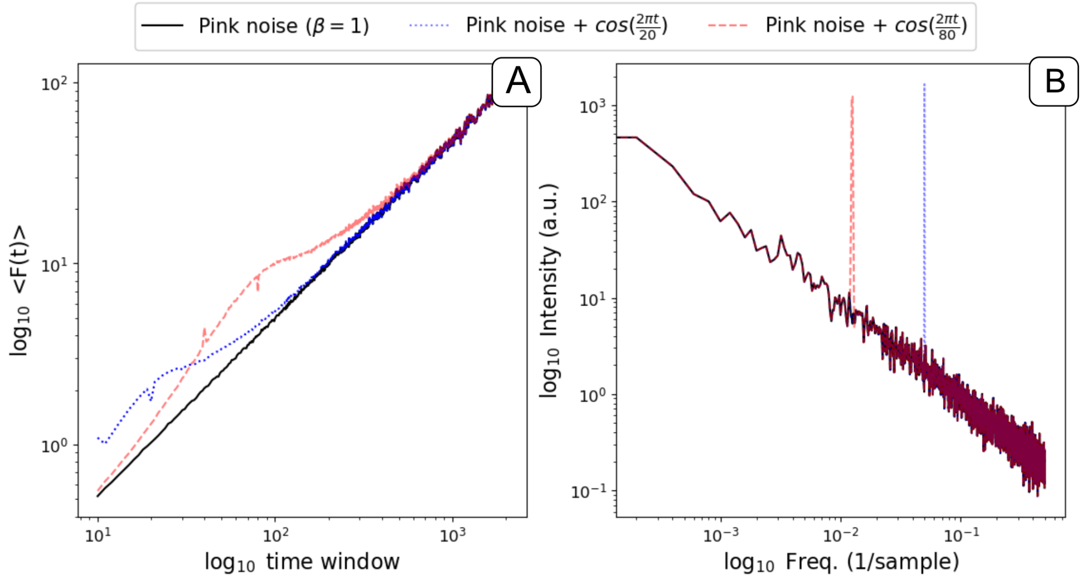

Supplementary Figure 6. Effects of periodic components on DFA and PSD. How periodic components affect the DFA fluctuation function and the PSD for signals showing long range correlatons was studied here by simulating pink noise (generated with the *colorednoise* Python package, mean = 0 and standard deviations 1) with an added cosine term of equal amplitude. A) DFA fluctuation function for different simulated signals; black line: pure pink noise (exponent $\beta = 1$), dotted blue line: pink noise with an additional cosine term of periodicity 20, dashed red line: pink noise with an added cosine term of amplitude 1 and periodicity 80. The presence of a periodic component leads to an additional broad shoulder centered around the periodicity on top of the otherwise pure power law. B) PSD estimation of the same simulated signals with the same color coding as in A). The addition of a periodic component leaves the PSD power law nearly unchanged apart from a sharp peak at the frequency of the component. This example demonstrates that for signals containing various periodic components, like musical time series (see Supplementary Figure 13), PSD analysis can be more meaningful than DFA.

Data

Classical music scores

The following is a list of all classical music pieces used in our analysis, sorted by composer. We used MIDI transcriptions mostly from the database kunstderfuge.com [7], but also from other databases [8, 9].

Single movements

J.S. Bach: Kunst der Fuge 07 (top voice), VIolin Sonata No.1 in G minor, BWV 1001, adagio; Violin Sonata No.1 in G minor, BWV 1001 presto; Gavotte; Chaconne (top notes); Partita for unaccompanied flute 1013, movements 1, 2, 3 & 4.
L. van Beethoven: String Trio in C minor, Op.9 No.3, movement 1 & 2; String Quartet No.13, Op.130, 1st movement; String Quartet No.6, Op.18 No.6, 1st movement; Symphony No.2, Op.36, 1st movement; Symphony No.2, Op.36, 3rd movement; Symphony No.1, Op.21, 3rd movement; Symphony No.4, Op.61, movements 1, 3 & 4; Violin Sonata No.4, Op.23 movements 1, 2 & 3; Violin Sonata No.8, Op.30 No.3, 3rd movement; Cello Sonata No.4, Op.102 No.1, 1st movement;
J. Brahms: String Quartet No.1, Op.51 No.1, 1st movement.
A. Dvorak: String Quartet No.10, Op.51, 1st movement; String Quartet No.11, Op.61, 1st movement; Serenade for Strings, Op.22, movements 1, 2, 3, 4 & 5.
J. Haydn: String Quartet in G major, Hob.III:41, 1st & 3rd movement; String Quartet in E-flat major, Hob.III:31, movement 1, 2, 3 & 4; String Quartet in D major, Op. 1 No. 3, Hob. III:3, 1st movement; String Quartet in G major, Op. 1 No. 4, Hob. III:4, 2nd movement.
D. Shostakovich: Prelude and Fugue No. 11 in B major (top voice); Prelude and Fugue No. 13 in F sharp major (top voice).
R. Schumann: String Quartet No.1, Op.41 No.1, movements 1 & 2.
W. Mozart:String Quartet No.17 in B-flat major, K.458, 3rd movement; String Quartet No.18 in A major, K.464 movements 1, 2, 3 & 4; Sonata for Violin and Piano in C major, KV 6, 1st movement.

Full compositions

J.S. Bach: Flute Sonata BWV 1013; Violin Sonata No.2 in A minor, BWV 1003; Violin Sonata No.1 in G minor, BWV 1001; Cello Suite No.1 in G major, BWV 1007; Prelude and Fugue in C-sharp major, BWV 848.
L. van Beethoven: String Trio in C minor, Op.9 No.3; Symphony No.2, Op.36; Symphony No.1, Op.21,, Violin Sonata No.4, Op.23 (top voice).
A. Dvorak: String Quartet No.10, Op.51; String Quartet No.11, Op.61; Serenade for Strings, Op.22
J. Haydn: Violin Sonata in D major; String Quartet in E flat major, Op. 20 No. 1, Hob. III:31; String Quartet Op. 33; String Quartet Op. 76.
W. Mozart: String Quartet No. 2 in D major, KV 155; Concerto for Piano and Orchestra No. 18 in B flat major, KV 456; String Quartet No. 20 in D major, KV 499; String Quartet No. 18 in A major, KV 464 ; String Quartet No. 14 in G major, KV 387; String Quartet No. 15 in D minor, KV 421; String Quartet No. 16 in E flat major, KV 428 (1783).
G. Handel: Violin Sonata in F major, HWV 370; Violin Sonata in A major, HWV 361; Violin Sonata in G minor, HWV 368; Violin Sonata in D major, HWV 371;
R. Schumann: Violin Sonata No.1, Op.105; Violin Sonata No.2, Op.121
P. Tchaikovsky: String Quartet No.1, Op.11
Wagner: Symphony in C.
A. Vivaldi: Trio Sonata “La Folia”.

Jazz Improvisations

As mentioned in the materials section, all the improvised music data that we used were obtained from the Weimar Jazz Database [10]. We removed pieces that were too short (less than 500 data points) or non-standard MIDI files. The following is the complete list of pieces we used sorted by artist.

Art Pepper: Stardust-1, Stardust-2
Benny Carter: I Got It Bad, It's a Wonderful World-1
Ben Webster: Did You Call Her Today
Bob Berg: I Didn't Know What Time It Was, Nature of The Beast, Angels.
Branford Marsalis: Gut Bucket Steepy, Housed From Edward-1, The Nearness of You, Three Little Words
Chris Potter: Arjuna, D.I.T., PopTune#1, Rumples, Togo
Clifford Brown: A Night In Tunisia, Jordu
Coleman Hawkins: Body and Soul, Sophisticated Lady
Curtis Fuller: Blue Train
David Liebman: Nica's Dream, Pablo's Story, Softly as In A Morning Sunrise, There Will Never Be Another You
David Murray: Blues for Two-1
Dexter Gordon: Society Red, The Rainbow People
Don Byas: Body and Soul
Eric Dolphy: Dahomey Dance
Freddie Hubbard: Dolphin Dance, Maiden Voyage
Hank Mobley: Remember, Soul Station
JJ Johnson: Walkin', Yesterdays
Joe Henderson: In'N Out-1, The Sidewinder, Totem Pole
Joe Lovano: Body and Soul-1, Central Park West, I Can't Get Started, Little Willie Leaps in
John Coltrane: Blue Train, Body and Soul – Alternate Take, Impressions - 1961, Impressions - 1963, My Favorite Things-1, My Favorite Things-2, Nature Boy, Nutty, So What
Kenny Dorham: Punjab
Lee Morgan: Blue Train
Michael Brecker: Confirmation, Midnight Voyage, Naked Soul
Miles Davis: Miles Runs the Voodoo Down-1. Miles Runs the Voodoo Down-2, Walkin'
Milt Jackson: Softly as in a Morning Sunrise
Ornette Coleman: Peace
Pat Metheny: All the Things You Are
Pepper Adams: A night In Tunisia
Sidney Bechet: Summertime
Sonny Rollins: Blue Seven-1, Vierd Blues
Stan Getz: Body and Soul, Crazy rhythm
Steve Coleman: The oracle-1, The oracle-2
Steve Lacy: Let's cool One
Steve Turre: Dat Dere
Wayne Shorter: Eighty one, Footprints

Dependence of the cutoff time on movement length

The observation of a correlation between the total length of pieces and their cutoff times raises the question as to why that might be the case. It is very likely that repetitions of themes and motifs within a piece have an influence on the cutoff time, and longer pieces allow for the development of longer musical ideas. Themes usually comprise motifs and sub-motifs that cannot always be unambiguously characterized as their identification relies on somewhat subjective criteria (e.g. musical meaning, punctuation, cadence culmination, etc.) [11, 12]. Automatic musical structure and theme detection is a research question on its own that lies outside the scope of the current work [13, 14]. For this reason we did not conduct an in depth analysis of the relationship between cutoff times and the different lengths (sub-motif, motif, theme) at play within musical pieces. However, the longest possible theme within a musical piece is limited by the duration of the movement in which it exists. We therefore checked for the influence of the structure associated with movements in classical compositions on the cutoff times. The results are shown in Supplementary Figure 7, where the cutoff times identified in pieces from various composers are plotted against the average movement length in the corresponding pieces. We observe no trend or correlation between average movement lengths and cutoff times, indicating that the cutoff is probably the result of the interplay between different melodic motifs present within the themes expressed in the various movements. The fact that this simple check shows no clear trend suggests that a profound investigation of the relationship between motif and theme lengths and cutoff times would not reveal any correlation.

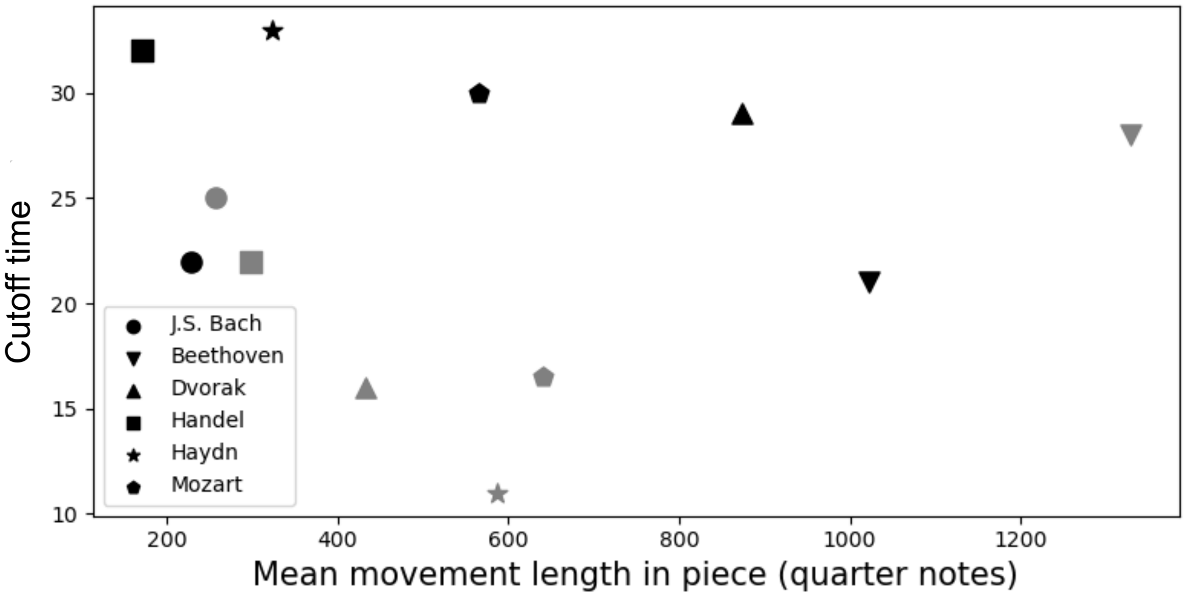

Supplementary Figure 7. Dependence of the cutoff time on the average movement length. Each dot represents a full composition from different composers and depicts the cutoff time as a function of the average length of the movements in the corresponding pieces. The color coding (black/gray) helps distinguishing pieces of the same composer. The pieces shown are the following, ordered by composers: J.S. Bach: Flute Sonata BWV 1013 (black); Cello Suite No.1 in G major, BWV 1007 (gray). L. van Beethoven: Symphony No.2, Op.36 (black); Symphony No.1, Op.21 (gray). A. Dvorak: String Quartet No.10, Op.51 (black); Serenade for Strings, Op.22 (gray). J. Haydn: Violin Sonata in D major (black); String Quartet in E flat major, Op. 20 No. 1, Hob. III:31 (gray); W. Mozart: String Quartet No. 14 in G major, KV 387 (black); String Quartet No. 15 in D minor, KV 421 (gray); G. Handel: Violin Sonata in F major, HWV 370 (black); Violin Sonata in A major, HWV 361 (gray)

Example for different average cutoff times depending on composer

In the main text of this article, we mentioned that the style of a composer may also be reflected in the cutoff times. In Supplementary Figure 8, we show such an example where we compare the cutoff times observed in movements by J.S. Bach to those in movements by Mozart. The movements from this corpus indicate that movements by Mozart on average tend to have longer cutoffs than those by Bach.

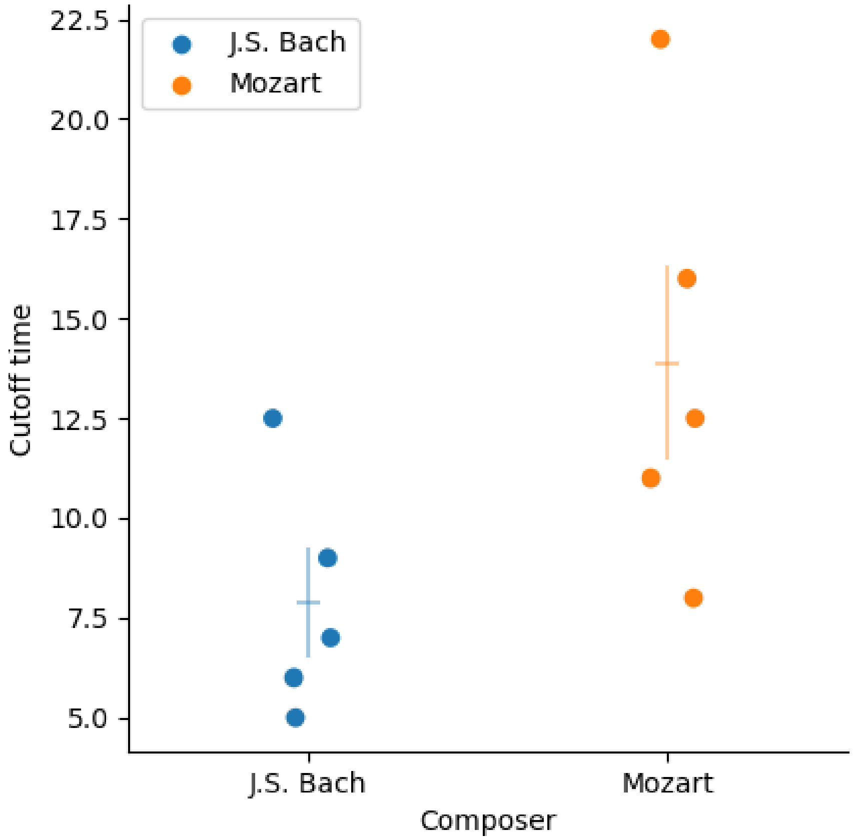

Supplementary Figure 8. Comparison of the cutoff time for single movements of J.S. Bach and Mozart. The blue points represent movements by J.S. Bach and the orange points movements by Mozart. Mean values and standard deviations are indicated by horizontal and vertical lines. Movements by Mozart tend to have longer cutoff times than those by J.S. Bach. For this plot, all movements of the corpus showing a PL+P were used. The pieces shown in this plot are the following: (J.S. Bach) Violin Sonata No.1 in G minor; Gavotte; Partita for unaccompanied flute 1013, movements 1, 2, & 4.; (Mozart) String Quartet No.17 in B-flat major, K.458, 3rd movement; String Quartet No.18 in A major, K.464 movements 1, 2 & 4; Sonata for Violin and Piano in C major, KV 6, 1st movement.

Rhythmic peaks: differences between classical and jazz

As stated in the main text of this article, the rhythmic peaks observed in classical compositions tend to be much stronger than in jazz improvisations. This can be seen in Supplementary Figure 9, where the distribution of the strongest identified rhythmic peaks in pieces is shown as a function of their intensity. While there is some overlap between the distribution for classical compositions and for jazz improvisations, rhythmic peaks are on average much stronger in classical compositions than in jazz improvisations. This difference can be explained by the fact that classical music tends to be rhythmically more regular than jazz, and the fact that jazz improvisers are known to play with micro-timing deviations [15, 16], both of which influence the strength of the periodicities identified by the power spectrum.

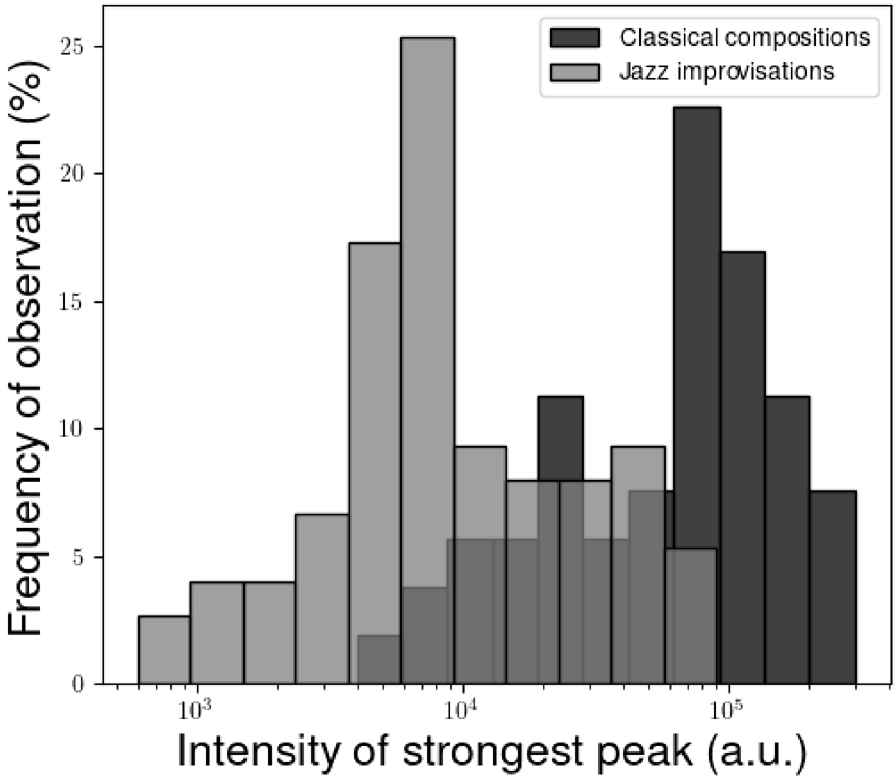

Supplementary Figure 9. Intensity of the strongest rhythmic peak identified in classical pieces and jazz improvisation. The light gray bars depict the proportion of observed strongest peaks as a function of their intensity in jazz improvisation. while the black bars show this relationship for classical compositions. For these histograms, only the strongest peak is extracted from each piece of the two datasets. While there is some overlap between the two distributions, we see that on average, rhythmic peaks are much stronger in classical compositions than in jazz improvisations.

Effects of slight plateau slopes on correlations

In some cases, weak slopes can be seen in the plateau part of the power spectra of jazz and classical pieces. Such trends can be attributed to a large variance in the low-frequency regime causing the fitting routine to identify slopes where there are none. Nevertheless, in some rare examples (e.g. see Supplementary Figure 14B), one might argue that these trends are not resulting from the fitting procedure and are indeed part of the musical time series. To account for such cases, and ensure the validity of our results, we ran several noise simulations allowing for a slight (positive and negative) slope in the plateau part of the corresponding power spectrum. To generate noise with the desired PSD shape (power law ending in a plateau with a slight slope), we multiplied the Fourier transform of white noise (generated via the function ramdom.rand from the *numpy* package) with a vector representing the shape of the PSD we wish to simulate (a weak slope in the plateau part and a negative slope in the power-law part). Then the inverse Fourier transform was computed to obtain the time series of the simulated noise. From these simulated noisy signals, we computed the autocorrelation function in order to reveal the effects of such slight plateau slopes. The results can be seen in Supplementary Figure 10 Overall, we observe that a slight slope in the plateau only has a very weak effect on the corresponding autocorrelation profile compared to a purely flat plateau.

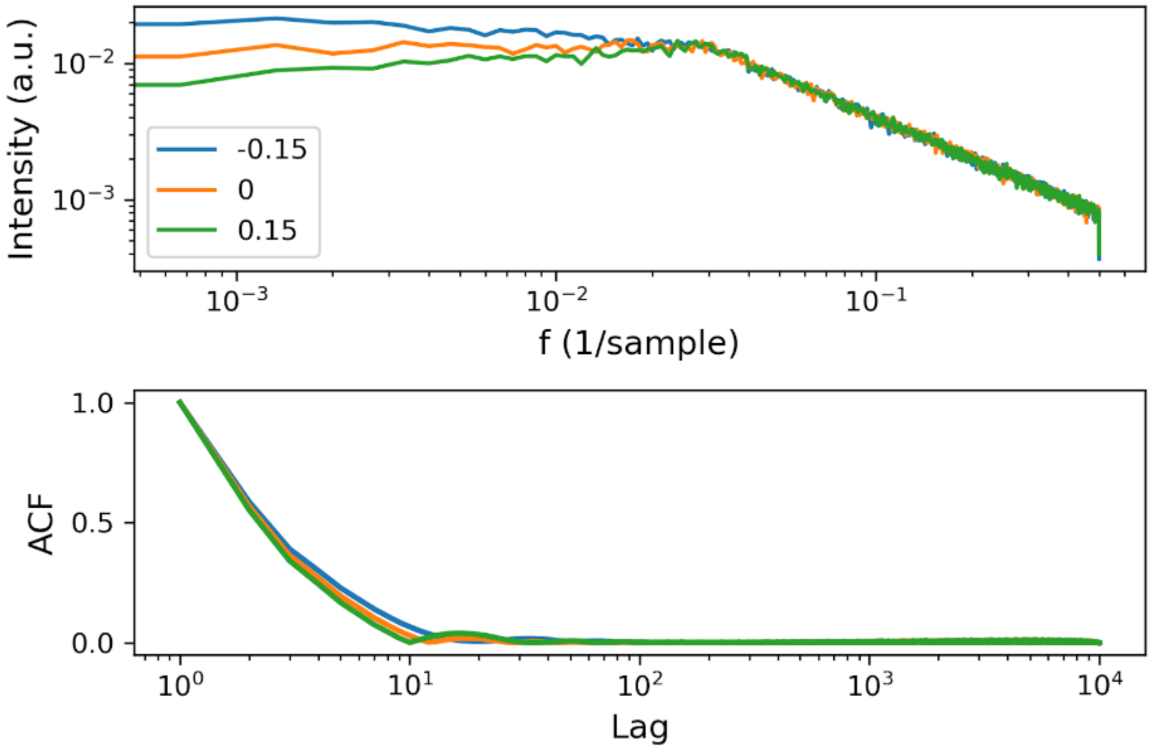

Supplementary Figure 10. Influence of weak slope in the plateau. To mimic and study the effects of the weak slopes observed in the plateaus of some rare example, signals showing a power-law and a weak slope at frequencies lower than the cutoff time in their PSD were simulated. In the top panel, each colored line represents the average PSD of 5 simulated PL+P signals having a weak indicated in the legend. The legend shows the slope of the plateau part of the PSD in the low frequency regime. In the botton pannel, the corresponding averaged autocorrelation function is displayed. We see that, overall such weak slopes do not have a considerable effect on the autocorrelation function, and the correlation decay is not strongly affected.

Cutoff time vs. power-law exponent

To check whether there is a correlation between the cutoff time of a musical piece and the exponent β, Supplementary Figure 11 shows scatter plots for the different datasets, in which the cutoff time of each piece is plotted against the exponent of the power-law part. In these plots only pieces are represented that show a PL+P shape. There is no indication of a correlation between these two variables.

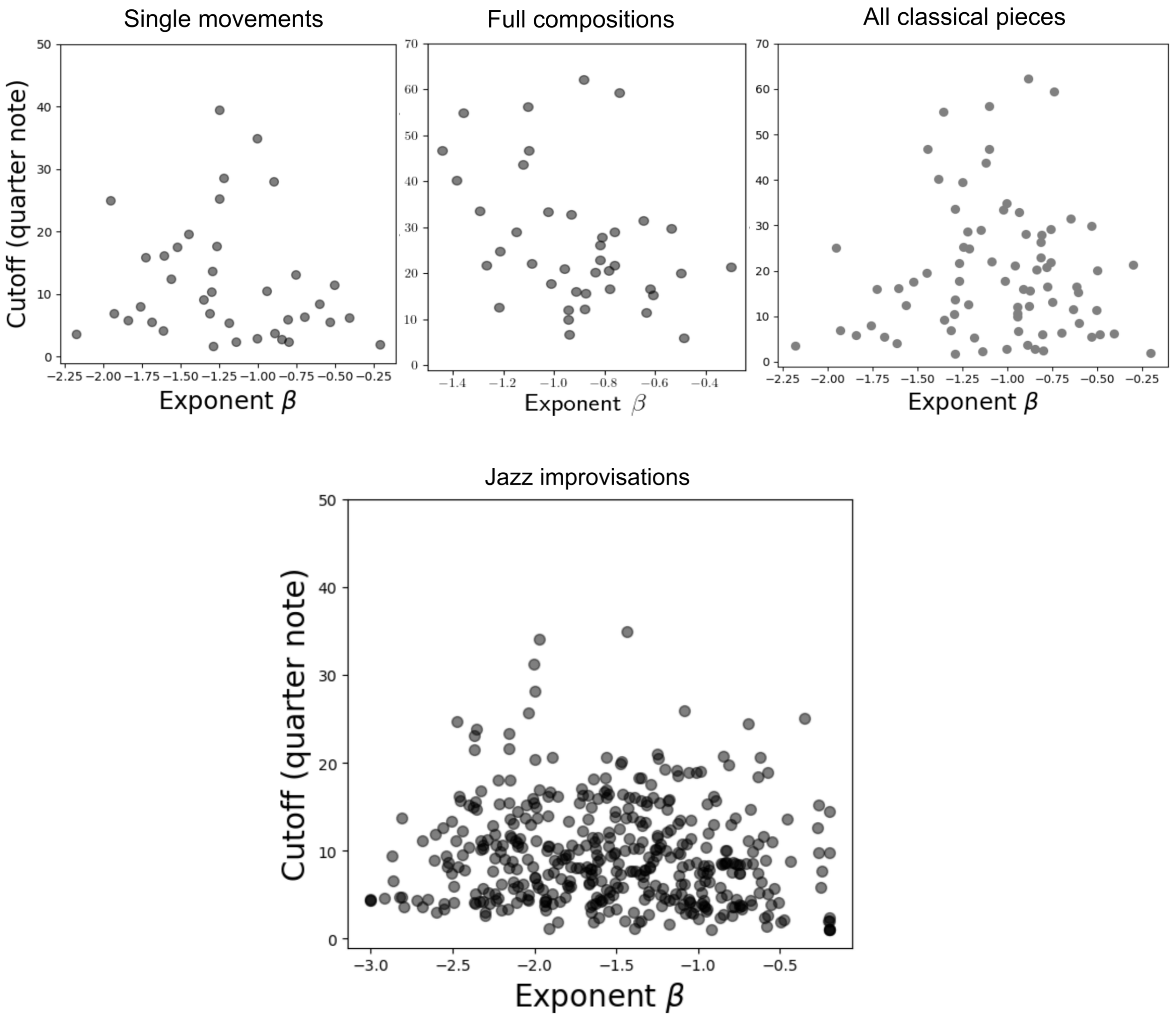

Supplementary Figure 11. Possible correlation between the cutoff time and the power law exponent β. Each point represents a piece in the different datasets relating the exponent β of the power-law part of the spectrum to the cutoff time. There appears to be no correlation between these two variables.

Power-spectral densities of additional examples

We carried out PSD analyses for a total of 553 musical pieces. The following Supplementary Figure 12, 13 and 14 show examples in addition to the examples in the main text for single movements, full compositional works, and improvised jazz solos.

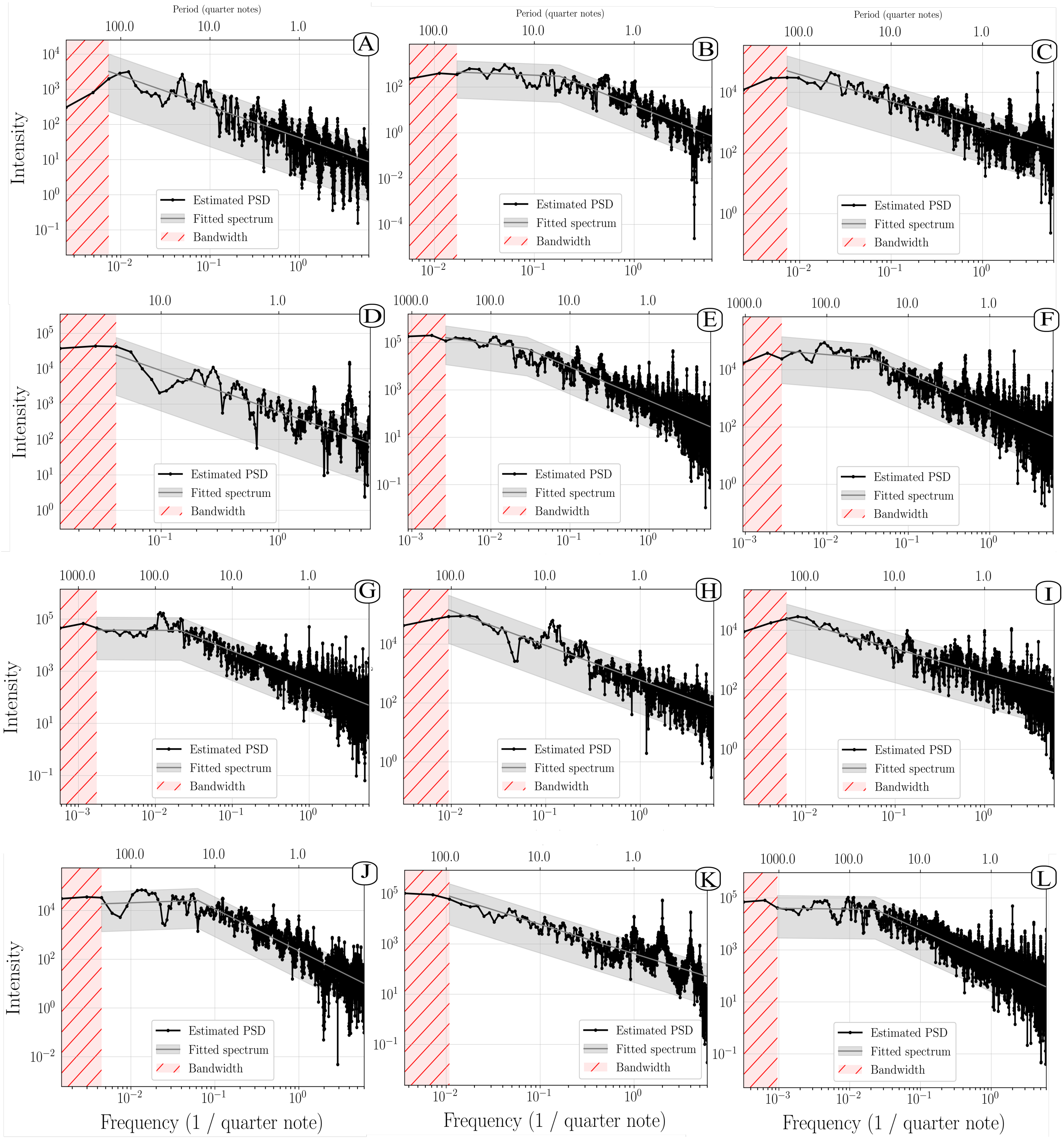

Supplementary Figure 12. PSD estimations of single movements. (A) Kunst der Fuge, BWV 1080 (Bach, Johann Sebastian); (B) Sarabande from Partita in A minor, BWV 1013 (Bach, Johann Sebastian); (C) Presto from Violin Sonata No.1 in G minor, BWV 1001 (Bach, Johann Sebastian); (D) Adagio molto from Symphony No.2, Op.36 (Beethoven, Ludwig van) (E) Presto from Violin Sonata No.1 in G minor, BWV 1001 (Bach, Johann Sebastian); (F) Tio in D major from 3 String Trios, Op.9 (Beethoven, Ludwig van); (G) Moderato from Serenade for Strings, Op.22 (Dvořák, Antonín); (H) Larghetto from Serenade for Strings, Op.22 (Dvořák, Antonín); (I) Allegro from String Quartet No.11, Op.61 (Dvořák, Antonín); (J) Andante from String Quartet No.18 in A major, K.464 (Mozart, Wolfgang Amadeus); (K) Allegro from String Quartet No.18 in A major, K.464 (Mozart, Wolfgang Amadeus); (L) String Quartet in E-flat major, Hob.III:31 from String Quartets, Op.20 (Haydn, Joseph).

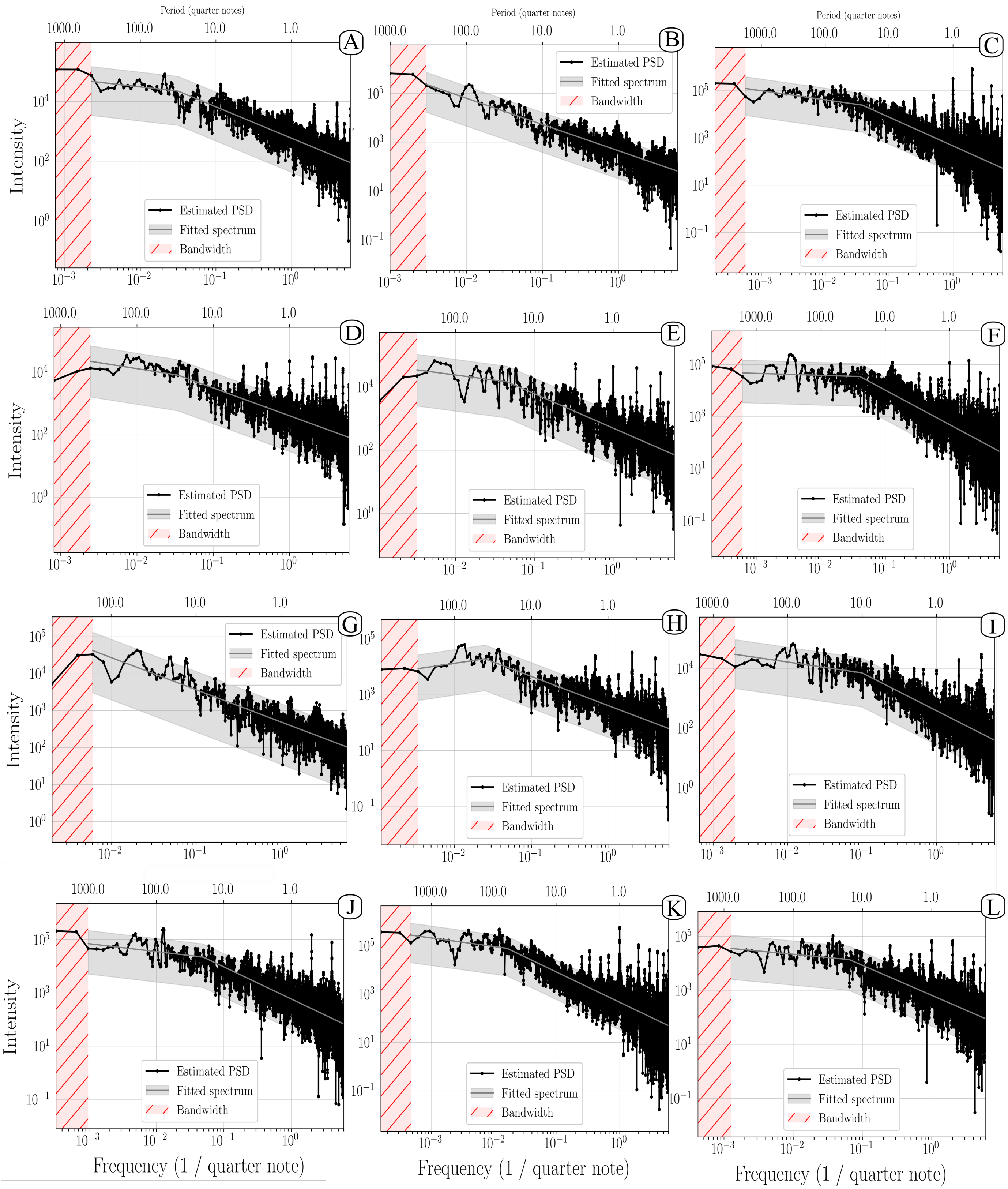

Supplementary Figure 13. PSD estimations of full compositional works. (A) Partita in A minor, BWV 1013 (Bach, Johann Sebastian); (B) Violin Sonata No.2 in A minor, BWV 1003 (Bach, Johann Sebastian); (C) Cello Suite No.1 in G major, BWV 1007 (Bach, Johann Sebastian); (D) String Trio in C minor, Op.9 No.3 (Beethoven, Ludwig van); (E) Symphony No.1, Op.21 (Beethoven, Ludwig van); (F) Symphony No.2, Op.36 (Beethoven, Ludwig van); (G) Serenade for Strings, Op.22 (Dvořák, Antonín); (H) Violin Sonata in A major, HWV 361 (Handel, George Frideric); (I) Violin Sonata in G minor, HWV 368 (Handel, George Frideric); (J) Sonata for Violin No.2 (Haydn, Joseph); (K) String Quartets, Op.20 (Haydn, Joseph); (L) String Quartet No.2 in D major, K.155/134a (Mozart, Wolfgang Amadeus).

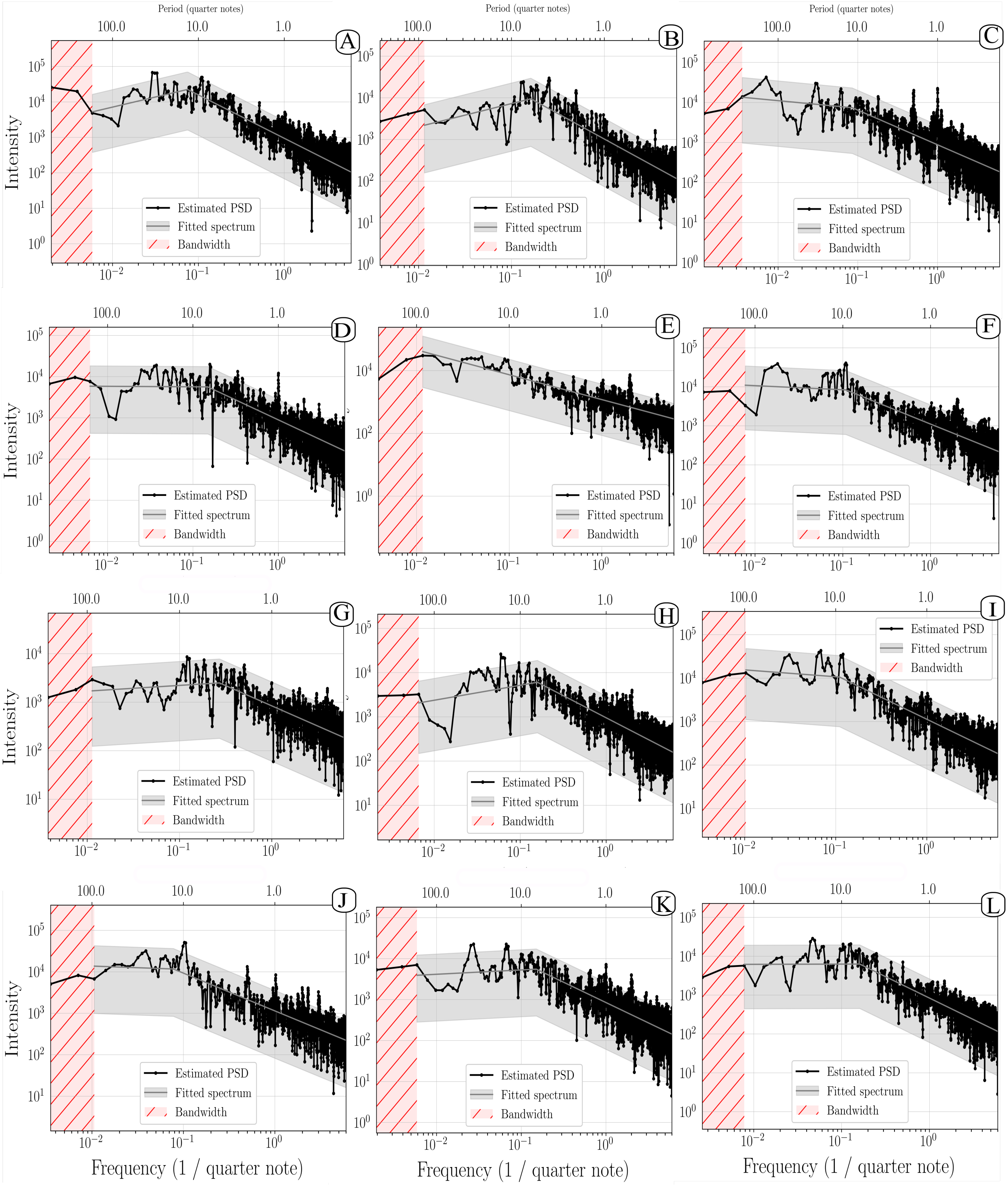

Supplementary Figure 14. PSD estimations of various jazz solos from the Weimar Jazz database [10]. (A) Anthropology (Art Pepper); (B) Stardust (Art Pepper); (C) Just Friends (Benny Carter); (D) It's a wonderful world (Benny Carter); (E) Did you call her today (Ben Webster); (F) When or where (Ben Webster); (G) Angels (Bob Berg); (H) Nature of the beast (Bob Berg); (I) House from Edward (Branford Marsalis); (J) U.M.M.G (Branford Marsalis); (K) Arjuna (Chris Potter), (L) Blues for two - take 1- (David Murray).

Supplementary References

[1] Nigel Nettheim. On the spectral analysis of melody. *Interface*, 21(2):135–148, 1992.

[2] Alfredo González-Espinoza, Hernán Larralde, Gustavo Martínez-Mekler, and Markus Müller. Multiple scaling behaviour and nonlinear traits in music scores. *Royal Society Open Science*, 4(12):171282, dec 2017.

[3] C-K Peng, Shlomo Havlin, H Eugene Stanley, and Ary L Goldberger. Quantification of scaling exponents and crossover phenomena in nonstationary heartbeat time series. *Chaos: An Interdisciplinary Journal of Nonlinear Science*, 5(1):82–87, 1995.

[4] Richard Hardstone, Simon Shlomo Poil, Giuseppina Schiavone, Rick Jansen, Vadim V. Nikulin, Huibert D. Mansvelder, and Klaus Linkenkaer-Hansen. Detrended fluctuation analysis: A scale-free view on neuronal oscillations. *Frontiers in Physiology*, 3 NOV, 2012.

[5] D. Marković and M. Koch. Sensitivity of hurst parameter estimation to periodic signals in time series and filtering approaches. *Geophysical Research Letters*, 32(17), 2005.

[6] Radhakrishnan Nagarajan and Rajesh G. Kavasseri. Minimizing the effect of periodic and quasi-periodic trends in detrended fluctuation analysis. *Chaos, Solitons and Fractals*, 26(3):777 – 784, 2005.

[7] Terry Smythe et al. Kunstderfuge www.kunstderfuge.com (2002)

[8] Metronimo https://www.metronimo.com (1999)

[9] Casey A. Mullin. International music score library project. *Notes*, 67(2):376–381, 2010.

[10] Martin Pfleiderer, Klaus Frieler, Jakob Abeßer, Wolf-Georg Zaddach, and Benjamin Burkhart, editors. *Inside the Jazzomat - New Perspectives for Jazz Research.* Schott Campus, 2017.

[11] Jean-Jacques Nattiez. *Music and discourse: Toward a semiology of music.* Princeton University Press, 1990.

[12] Bruce Benward. *Music in theory and practice vol. 2.* London, United States: McGraw Hill Higher Education, 2018.

[13] Namunu C Maddage. Automatic structure detection for popular music. *Ieee Multimedia*, 13(1):65–77, 2006.

[14] Joan Serra, Meinard Müller, Peter Grosche, and Josep Ll Arcos. Unsupervised music structure annotation by time series structure features and segment similarity. *IEEE Transactions on Multimedia*, 16(5):1229–1240, 2014.

[15] George Datseris, Annika Ziereis, Thorsten Albrecht, York Hagmayer, Viola Priesemann, and Theo Geisel. Microtiming deviations and swing feel in jazz. *Scientific Reports*, 9(1):19824, 2019.

[16] Corentin Nelias, Eva Marit Sturm, Thorsten Albrecht, York Hagmayer, and Theo Geisel. Downbeat delays are a key component of swing in jazz. *Communications Physics*, 5(1):237, 2022.
